# Beam Hardening Artifacts: Comparison between Two Cone Beam Computed Tomography Scanners

**DOI:** 10.5681/joddd.2012.011

**Published:** 2012-06-06

**Authors:** Farzad Esmaeili, Masume Johari, Pezhman Haddadi, Mehdi Vatankhah

**Affiliations:** ^1^Assistant Professor, Department of Oral & Maxillofacial Radiology, Faculty of Dentistry, Tabriz University of Medical Sciences, Tabriz, Iran; ^2^Post-graduate Student, Department of Oral & Maxillofacial Radiology, Faculty of Dentistry, Tabriz University of Medical Sciences, Tabriz, Iran; ^3^Department of Oral & Maxillofacial Radiology, Faculty of Dentistry, Tabriz University of Medical Sciences, Tabriz, Iran

**Keywords:** Artifacts, beam hardening, cone beam computed tomography

## Abstract

**Background and aims:**

At present, cone beam computed tomography (CBCT) has become a substitute for computed tomography (CT) in dental procedures. The metallic materials used in dentistry can produce artifacts due to the beam hard-ening phenomenon. These artifacts decrease the quality of images. In the present study, the number of artifacts as a result of beam hardening in the images of dental implants was compared between two NewTom VG and Planmeca Promax 3D Max CBCT machines.

**Materials and methods:**

An implant drilling model was used in the present study. The implants (Dentis) were placed in the canine, premolar and molar areas. Scanning procedures were carried out by two CBCT machines. The corresponding sections (coronal and axial) of the implants were evaluated by two radiologists. The number of artifacts in each image was determined using the scale provided. Mann-Whitney U test was used for two-by-two comparisons at a significance level of P<0.05.

**Results:**

There were statistically significant differences in beam hardening artifacts in axial and coronal sections between the two x-ray machines (P<0.001), with a higher quality in the images produced by the NewTom VG.

**Conclusion:**

Given the higher quality of the images produced by the NewTom VG x-ray machine, it is recommended for imaging of patients with extensive restorations, multiple prostheses or previous implant treatments.

## Introduction

Cone beam computed tomography (CBCT) is a very important radiographic technique to diagnose head and neck region lesions. It has become an important diagnostic tool in dentistry in recent years. Use of CBCT in dental procedures has increased in recent years due to its low cost, fast image production rate and its low radiation dose in comparison with CT. CBCT appears to have a high potential in the diagnosis and treatment planning, especially in implant treatments, by providing three-dimensional images.^1 - 4^

If a metal is present in the area to be scanned, the images are prone to production of artifacts. Artifact is any distortion or error in the image that is unrelated to the subject. Artifact is the main cause of decrease in image quality and in some cases the artifact render the image useless.^4^ Some of these artifacts are produced due to a phenomenon, referred to as beam hardening. When the x-ray beam travels through an object, the low-energy photons are absorbed more than the high-energy photons; this phenomenon is referred to as beam hardening. This phenomenon is produced by objects with a high density.^4 - 7^

Since CBCT uses back-projected beams to produce three-dimensional images and its image production principles are similar to those of CT, these artifacts can also be produced in the CBCT images, too.^4 , 5^

Although there are many techniques to reduce the number of these artifacts in CT technique ^8- 14^ only a limited number of techniques have been introduced to counteract these artifacts in the CBCT technique.^15 , 16^ In the clinic, it has been suggested to decrease the field of view, change the position of patient head or separate dental arches in order to avoid scanning the areas susceptible to beam hardening.^5^ It seems that the type of the machine is also effective in producing artifacts, although only a limited number of studies have been carried out in this issue. ^2- 4^

Exposure conditions can have a great role in producing artifacts by influencing the energy of the photons; some studies have recommended imaging techniques with high kVp to decrease hardening of the beams.^1 , 4^ Other factors that can have a role in beam hardening include the amount of rotation of the machine, the configuration of the x-ray beam and the type of the algorithm used for data processing.^17 - 19^

A large number of previous studies have evaluated metallic artifacts in CBCT images;^1 , 4 , 6 , 15^ however, the majority of these studies have been qualitative ones. A few studies have quantitatively compared CBCT x-ray machines.^1 , 17^ Given the paucity of studies, particularly studies comparing CBCT x-ray machines with each other, studies appear to be necessary in this respect.

The aim of the present study was to compare the artifacts produced by Planmeca Promax 3D Max and NewTom VG CBCT machines as a result of beam hardening phenomenon during scanning of dental implants.

## Materials and Methods

A dry human skull was used in the present study. Since the aim of the present study was to evaluate artifacts produced by beam hardening in dental implants without interference of any other materials, an implant drilling model (Nissian, Kyoto, Japan), which is completely similar to a human mandible, was used instead of human mandible.^17^

**Figure 1:**
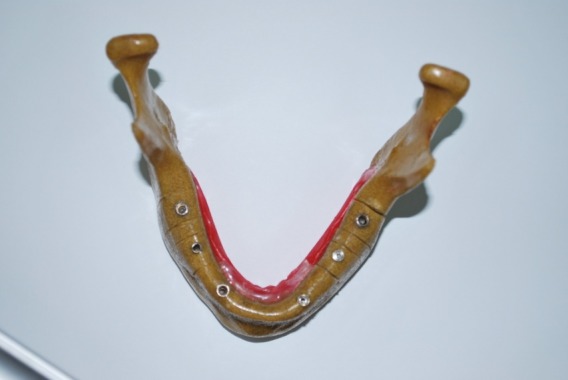
The implant drilling model used in the study.

Dentis implant system (Dentis, Daegu, Korea) was used to evaluate artifacts. Two implants were placed in the canine area, two in the second premolar area and two in the second molar area (Figure 1). The implants measured 12 mm in length and 4.3 mm in diameter. On the whole, three series of scans (canine, premolar and molar) were carried out by NewTom VG CBCT (QR SRL Company,Verona, Italy) and Planmeca Promax 3D Max cone beam CT (Planmeca OY, Helsinki, Finland). Gutta-percha was used as a marker to determine axial and coronal section locations. In each implant site, identical sections were selected from axial and coronal sections.

The first series of scans were carried out by NewTom VG x-ray machine in the Department of Oral and Maxillofacial Radiology, Faculty of Dentistry, Tabriz University of Medical Sciences. This x-ray machine uses a cone x-ray beam with 1920×1536 pixel flat panel detector, a rotation of 360º, a pixel size of 0.127 μm and 120 kVp. The scans were carried out at kVp110 kVp, exposure time of 3.6 seconds and 4.71 mA. Initial and final reconstruction was carried out by NNT Viewer software version 2.21 (Quantitative Radiology, Verona, Italy).

The second series of scans were carried out by Planmeca Promax 3D Max CBCT (Planmeca OY, Helsinki, Finland) in a private oral and maxillofacial radiology clinic in Tabriz. This x-ray machine uses a cone x-ray beam, with 1900×1516 pixel flat panel detector, a rotation of 270º, a pixel size of 0.127 μm and a 84 kVp. The scans were carried out under the following exposure conditions: kVp84 kVp, exposure time of 12 seconds and 12 mA.

Initial and final reconstructions were carried out by Romexis 2.3.1 software (Planmeca, Helsinki, Finland).

**Figure 2:**
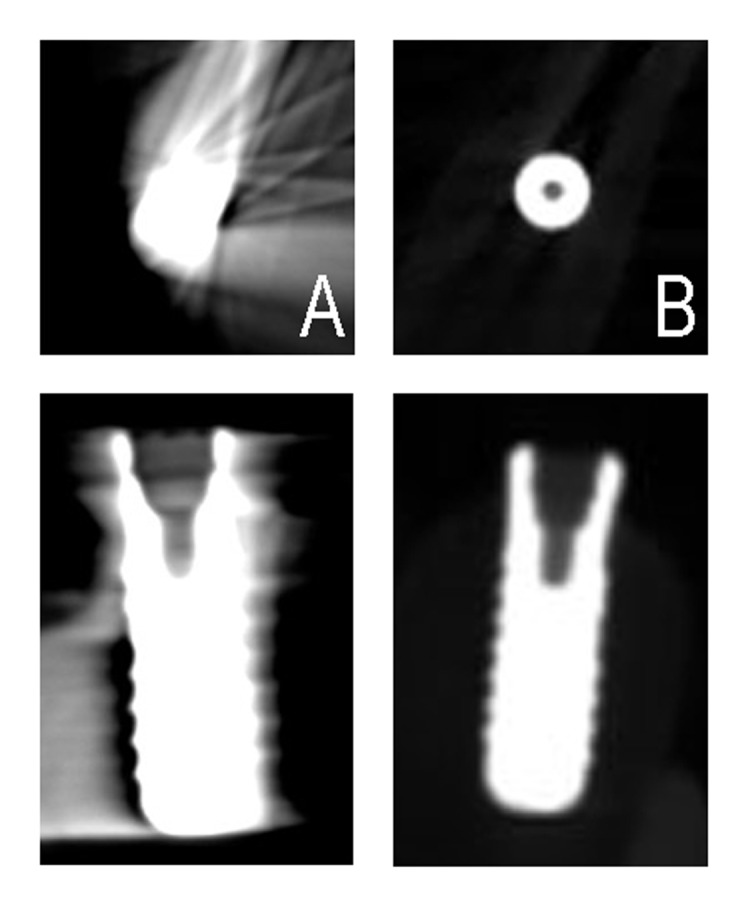
(A) Planmeca Promax 3D Max CBCT images (Axial and Coronal ; Molar region). (B) NewTom VG CBCT images (Axial and Coronal; Molar region).

Three equal sets of images from two cone beam CT scanner were evaluated by three independent observers, who were oral and maxillofacial radiologists each with more than 4 years of experience in the analysis of CBCT scans. Kappa statistics was used for evaluating inter-observer agreement. There was substantial agreement between observers. Images as demonstrated in Figure 2, which includes axial and coronal displays, were presented to the observers. The images were evaluated on a 17 inch monitor (cathode ray tube) of a desk-top computer. The evaluation was carried out in a windowless room under mild lighting conditions. A standardized rating was used for this quality evaluation (Table 1).

**Table 1:** The image quality assessment evaluation rating^17^

Rating	Description
5 4 3 2 1	No beam hardening artifacts Minimal beam hardening artifacts: more than 90% of the implant structure is imaged correctly Moderate beam hardening artifacts: more than 75% of the implant structure is imaged correctly Strong beam hardening artifacts: more than 50% of the implant structure is imaged correctly Severe beam hardening artifacts: less than 50% of the implant structure is imaged correctly

Mann-Whitney U test was used for two-by-two comparisons, when there were statistically significant differences, using SPSS 13 statistical software (SPSS Inc, Chicago, USA). Statistical significance was defined at P<0.05.

## Results

In the present study three series of scans (canine, premolar and molar) were carried out by two CBCT x-ray machines. In the axial section, the means of quality were 4.43 and 1.08 in the NewTom and Planmeca x-ray machines, respectively (Table 2). Mann-Whitney U Test revealed statistically significant differences in image quality between the two x-ray machines (P<0.001), with less artifacts and higher quality with the use of NewTom x-ray machine compared to Planmeca x-ray machine.

**Table 2:** The quality of images in the axial, coronal and both sections of NewTom VG (N) and Planmeca Promax 3D Max (P)

Section	Mean	SD	P value
Axial section			
N P	4.43 1.08	0.500 0.279	0.001
Coronal section			
N P	4.55 2.47	0.502 0.566	0.001
Both sections			
N P	4.49 1.78	0.502 0.825	0.001

P value of Mann-Whitney U test. SD: Standard deviation.

In the coronal section, the means of quality were 4.55 and 2.47 in the NewTom and Planmeca x-ray machines, respectively (Table 2). Mann-Whitney U test revealed statistically significant differences in image quality between the two x-ray machines (P<0.001), with less artifacts and therefore higher image quality with the use of NewTom x-ray machine compared to Planmeca x-ray machine.

In both sections, the means of quality were 4.49 and 1.78 in the NewTom and Planmeca x-ray machines, respectively (Table 2), with statistically significant differences between the two x-ray machines (P<0.001). NewTom x-ray machine yielded less artifacts and better quality; Planmeca x-ray machine yielded a higher number of artifacts in the coronal sections compared to the axial sections (Table 2). In both x-ray machines, the number of artifacts was higher in the axial sections compared to the coronal sections (Table 2).

## Discussion

CBCT is very valuable in the diagnosis and treatment planning in dentistry and medicine in the head and neck region. Therefore, the highest-quality images are necessary for treatment planing.^5 , 7^ Metallic restorations produce artifacts in the images produced by three-dimensional imaging systems. It becomes more important when the patient has extensive prostheses, amalgam restorations or implants in the oral cavity. The artifacts produced by metallic objects are the result of beam hardening phenomenon, which occurs in all the CBCT x-ray machines. ^4- 7^ Evaluation and comparison of different x-ray machines in relation to the extent of artifacts are very important because in some cases the artifacts are so extensive that image quality decreases or even the image is distorted.^5 - 7^

Schulz et al evaluated the image qualities of NewTom 900 and Siemens Siremobil scanners in a dry skull and reported no artifacts as a result of beam hardening, which was attributed to the fact that no metallic structures were used in the study.^18^ In the present study, different amounts of metallic artifacts were observed due to the use of titanium implants in both scanners under study.

Some researchers evaluated the metallic artifacts produced by dental metals with the use of Light Speed QX/I (MDCT) and Alpha Vega 3030 (CBCT) scanners. Cubes of aluminum, titanium, chromium-cobalt and Type IV gold alloy were scanned by the two scanners and the images were evaluated and compared by Image J software. The results showed fewer artifacts with CBCT scanners compared to MDCT scanners under identical conditions. In addition, increase in kVp in both scanners resulted in a decrease in artifacts. However, an increase in tube electric current had no effect on artifacts. An increase in kVp resulted in a decrease in beam hardening by influencing the energy of the photons.^1^

The results of the present study showed that NewTom VG x-ray machine (110 kVp) produces less artifacts compared to Planmeca Promax x-ray machine (84 kVp), which might be attributed to the higher kVp of NewTom VG.^1 , 4^In addition, the dental implants used in the present study produced severe metallic artifacts in a manner similar to the titanium blocks used in the above-mentioned study.

Shulze et al^4^ evaluated the artifacts produced by dental implants with the use of Accuitomo and 3D Exam CBCT scanners by studying the geometric and physical parameters effective on data collected from scans for the reconstruction of three-dimensional images. The results showed a great amount of artifacts produced by titanium implants under standard conditions. In the present study, also, titanium implants produced artifacts. In addition, the results of that study showed that scanning at higher kVp conditions results in fewer artifacts. Similarly, in the present study, NewTom VG x-ray machine exhibited fewer artifacts in comparison to Planmeca Promax, which might be due to its higher kVp.

Some authors compared the artifacts of dental implants with the use of NewTom 9000 (CBCT) and Philips MX 8000 (4-row MDCT). The axial and coronal images of implants in the canine and molar areas of the maxilla were compared in a model of skull made from saw bone material. The results showed much less artifacts with MDCT in comparison to CBCT, with the image of the implant in MDCT correctly produced in all the axial and coronal cross-sections in comparison to the main implant. Only 16% of the implants in MDCT had artifacts while the images of CBCT had no artifacts in less than 25% of cases. In addition, the results showed that there were more artifacts in the canine area compared to the molar area because of the position of canine in arch that made it vulnerable to more artifacts.^17^

In the present study, NewTom VG x-ray machine was used, which is newer and more advanced than the x-ray machine used in a study by Draenert, with a higher kVp (110 vs 86). Fewer artifacts were produced in comparison with the NewTom 9000 x-ray machine due to a higher kVp. On the other hand, Planmeca Promax x-ray machine exhibited artifacts similar to those produced by NewTom 9000 x-ray machine due to the similar kVp of 84. In addition, Draenert suggested further studies with kVp values >90 to decrease artifacts. In the present study, a higher kVp resulted in a decrease in artifacts, confirming the results reported by Draenert.

Metallic artifacts are similar to streak-shaped rays and interfere with details, especially in the transverse direction, i.e. those in the direction of x-ray beams. Therefore, these artifacts are produced in the direct direction in axial sections.^1 , 6 , 7 , 13^ In the present study, the artifacts were more numerous in axial sections in comparison with coronal sections, especially with Planmeca Promax x-ray machine.

In the present study, the number of artifacts with Planmeca Promax x-ray machine was significantly different from those produced by NewTom VG in all the sections, which might be attributed to a lower kVp in Planmeca Promax x-ray machine. Other involving factors might be the lower rotation of this unit (270º) and the type of software used in Planmeca Promax x-ray machine.^17 - 19^

## Conclusion

NewTom VG exhibited fewer beam hardening artifacts compared to Planmeca Promax 3D Max. Given the higher quality of images produced by NewTom VG x-ray machine, it is recommended for imaging producers in patients with extensive restorations, multiple prostheses or previous implant treatments.
